# Enhancing student creativity in AIGC design education: the mediating role of learning motivation in reverse engineering pedagogy

**DOI:** 10.3389/fpsyg.2026.1829753

**Published:** 2026-06-19

**Authors:** Gang Liu, Xu Zhang

**Affiliations:** 1 School of Media Arts and Communication, Nanjing University of the Arts, Nanjing, China; 2 Faculty of Innovation and Design, City University of Macau, Macau, China

**Keywords:** AIGC, creativity, design education, learning motivation, REP

## Abstract

**Introduction:**

The widespread application of artificial intelligence generated content (AIGC) technology in design education presents challenges in balancing technological dependency and autonomous innovation during creativity development. Establishing a dynamic equilibrium between technological empowerment and innovation capacity is critical in educational practice. This study examines how reverse engineering pedagogy (REP) stimulates learning motivation to foster creativity and proposes a multidimensional creativity evaluation framework.

**Methods:**

A quasi-experimental design with intact class-based quasi-random allocation was employed, involving 80 first-year animation majors from two intact classes (40 students per class) assigned to an experimental group (REP intervention) and a control group (traditional instruction). Quantitative assessments using multidimensional creativity and learning motivation scales, supported by statistical analysis, revealed inter-variable relationships.

**Results:**

The results indicated that the REP framework, comprising structural analysis, functional mapping, and creative reconstruction, significantly enhanced creative design thinking. Learning motivation partially mediated the effect of REP on creativity, confirming synergistic influences of intrinsic and extrinsic motivation.

**Discussion:**

The study proposes a multidimensional creativity evaluation framework integrating REP and learning motivation, and provides theoretical-practical guidance for pedagogical innovation in AIGC-driven design education.

## 1 Introduction

The rapid development of artificial intelligence generated content (AIGC) has reshaped educational practices, particularly in design education. AIGC tools (e.g., DALL·E, Midjourney, Stable Diffusion) enhance (Ladachart et al., 2022) students' creative efficiency by automating the generation of diverse design prototypes (Batista et al., 2024; Omran Zailuddin et al., 2024). Despite these advantages, educators have expressed concerns that excessive reliance on AIGC may reduce students' autonomous innovation capabilities (Park, 2024; Wang, 2021). Designing pedagogical interventions that balance technological assistance with creativity cultivation has therefore become a key research focus (Giannakos et al., 2024). However, AIGC design education differs fundamentally from traditional design pedagogy, necessitating a re-examination of established instructional models. In conventional design education, students typically engage in manual ideation and iterative sketching, with the cognitive load focused on translating abstract concepts into concrete visual forms. In contrast, AIGC tools externalize the generative act, shifting the student's role from direct production to prompt articulation, evaluative selection, and iterative refinement. This reconfiguration of the creative workflow alters both the cognitive demands placed on learners and the mechanisms by which creativity emerges. Creativity, the core objective of design education, involves solving complex problems by integrating novelty and practicality (Sekowski, 1999). Existing research largely examines either the direct effects of technological tool optimization on creativity or isolated pedagogical methods such as project-based learning. Few studies systematically integrate technological tools, teaching strategies, and psychological mechanisms (Bond et al., 2024; Koshanova et al., 2021). Creativity is not only essential for organizational adaptation and development but also crucial for students' continuous self-improvement and breakthroughs in learning (Vettorello et al., 2022). Therefore, enhancing creativity in AIGC-assisted design education has become a critical issue in contemporary educational research and practice.

Reverse engineering pedagogy (REP), an active learning strategy based on constructivist learning theory, has shown considerable potential in engineering education, STEM interdisciplinary curricula, and design thinking training (Ladachart et al., 2022; López et al., 2019; Zhong et al., 2024). REP engages students in deconstructing and analyzing existing design cases to deepen their understanding of design principles and processes, thereby fostering independent thinking and innovation (Fathoni, 2023). In AIGC education, however, REP has been applied mainly for technical guidance and skills training, with limited investigation into its indirect influence on creativity through psychological mechanisms (Paesano, 2021). This lack of research limits the comprehensive development of creativity and constrains the broader application of REP in AIGC education (Yuan and Liu, 2025).

**Figure 1:**
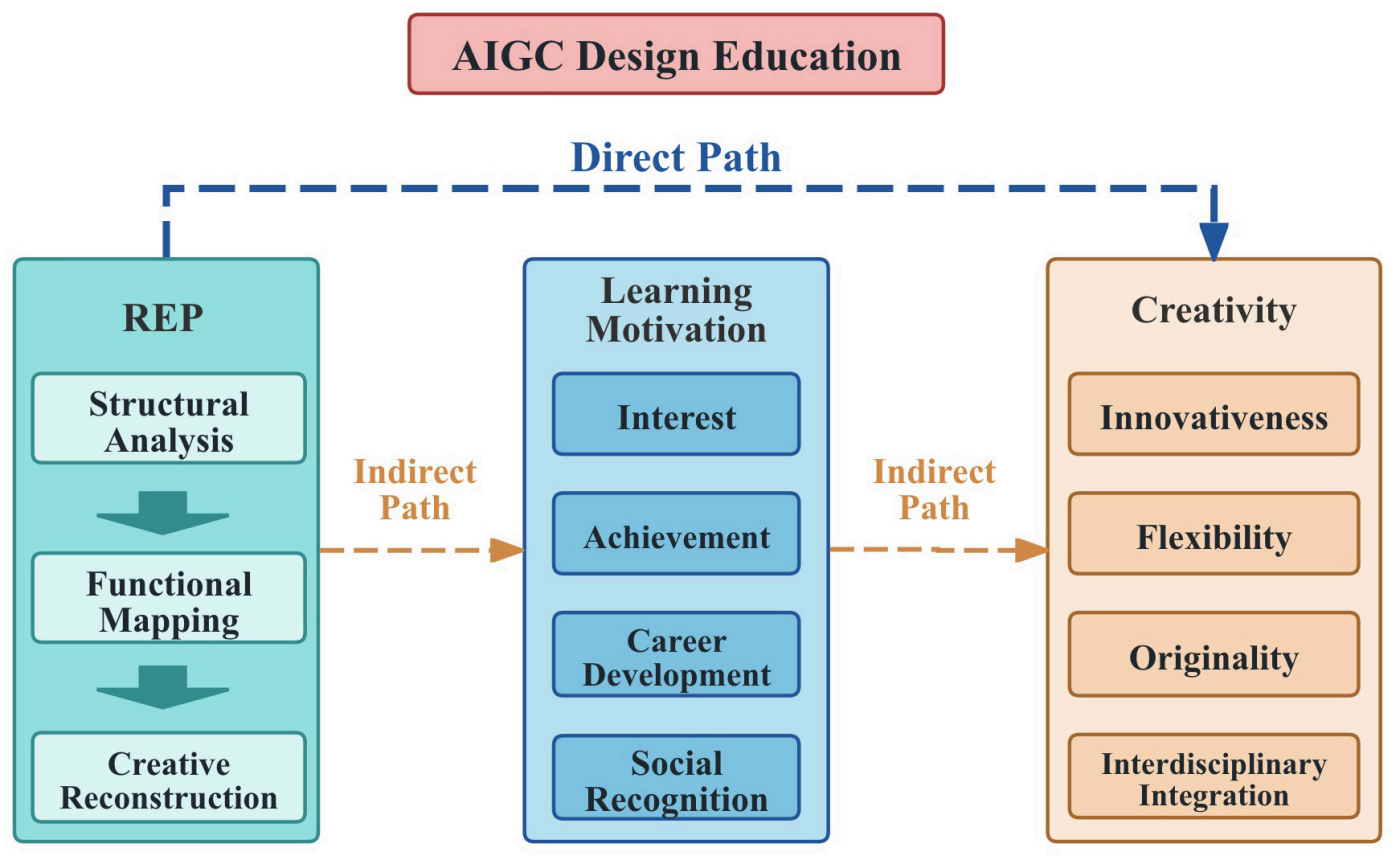
Conceptual framework of the proposed theoretical model.

Grounded in self-determination theory (SDT), this study examines how REP, as an instructional intervention, may enhance student creativity in AIGC design education through the mediating mechanism of learning motivation. SDT, which explains the motivational processes underlying self-determined behavior, has been widely applied in education and shown to significantly influence student motivation and creativity (Deci and Ryan, 2008). The theory highlights the balance between intrinsic and extrinsic motivation, which directly affects learning attitudes and performance (Ryan and Deci, 2020). A key question addressed in this study is how instructional design in AIGC-assisted education can strengthen intrinsic motivation to enhance creativity. The integration of REP with learning motivation principles to examine its effects on creativity remains underexplored (Kuleto et al., 2021). Therefore, identifying teaching strategies that stimulate learning motivation and, in turn, improve creativity is a pressing issue in AIGC design education (Lua et al., 2024). This study investigates how REP can enhance creative performance by stimulating learning motivation based on SDT. By constructing a multidimensional creativity evaluation framework, it seeks to clarify how the combined application of learning motivation theory and REP interventions can improve creative outcomes, providing theoretical and practical guidance for AIGC-assisted design education. The theoretical model underpinning this research is shown in Figure 1. Accordingly, the study addresses the following central research question: “How does REP enhance student creativity by stimulating learning motivation within AIGC design education, thereby enabling the construction of a multidimensional creativity evaluation framework?” Three sub-questions guide the investigation:

1) In AIGC design education, does the REP pedagogical intervention significantly enhance students' creativity?
2) Does learning motivation (interest, achievement, career development, and social recognition) play a mediating role between REP and creativity enhancement in AIGC design education?
3) How can a multidimensional creativity evaluation framework suitable for AIGC design education be proposed based on REP and learning motivation?

## 2 Literature review

### 2.1 AIGC design education and creativity

AIGC tools reshape design education by automating routine tasks, expanding creative boundaries, and providing real-time feedback (Auernhammer, 2020; Hwang and Wu, 2025). Despite these transformative benefits, widespread AIGC adoption brings a core unresolved pedagogical challenge: over-reliance on AIGC may reduce students' active cognitive engagement, discourage independent ideation, and undermine their design agency and creative autonomy, resulting in homogeneous, unoriginal design outputs lacking personalization and innovative depth (Baltà-Salvador et al., 2025; Xu et al., 2024), making the optimal balance between AI technical assistance and student autonomous creativity a critical priority in contemporary design education.

To address this tension, scholars have explored integrating AIGC with established pedagogical frameworks. Collaborative and inquiry-based learning models combined with AIGC tools enhance creativity and critical thinking (Kumar, 2021; Shahzad et al., 2025). From a motivational perspective, SDT posits that autonomy-supportive tasks stimulate intrinsic motivation, a key driver of creativity (Deci and Ryan, 2000); AIGC's personalized feedback heightens perceived autonomy and leads to more original outputs (Xia et al., 2022). Social Cognitive Theory complements this by demonstrating that AIGC expands design possibilities, elevating creative self-efficacy (Bandura, 1986; Tierney and Farmer, 2002). However, existing research has not yet identified a systematic instructional intervention that strategically leverages AIGC to maximize creativity while preserving students' autonomous creative agency, the core gap addressed in this study.

In summary, while existing research has separately documented AIGC's potential to enhance creative efficiency and its associated risk of fostering cognitive dependence, few studies have systematically examined how specific pedagogical interventions can strategically leverage AI tools while mitigating over-reliance. The mechanisms through which AIGC reshapes creative cognition, particularly the shift from generative to evaluative thinking, remain underexplored as a boundary condition for instructional design. This study addresses this gap by positioning AIGC not merely as a technological backdrop but as a contextual factor that fundamentally conditions REP's effectiveness.

### 2.2 Reverse engineering pedagogy

REP has evolved from its origins in industrial deconstruction into a recognized instructional strategy in engineering and design education (Wiesen et al., 2018). Its core educational premise that systematic analysis of existing artifacts cultivates deeper understanding and transferable innovation skills has been extended in recent scholarship to encompass digital and algorithmic systems (Shailendra et al., 2024). Of particular relevance to this study is the application of REP to AIGC outputs, where the pedagogical focus shifts from physical disassembly to the reverse analysis of generative logic and prompt-to-image relationships.

Reverse-engineering AIGC outputs allows exploration of latent design spaces, by analyzing stylistic features, parameter configurations, and semantic relationships, students deduce generative models' decision logic, transforming implicit machine knowledge into explicit design rules (Komolafe et al., 2024). This establishes a cyclical learning process of generation, analysis, and recreation. When AI outputs deviate from expectations, learners experience cognitive disequilibrium that stimulates creative cognition and reorganization of knowledge frameworks (Cascini et al., 2022). The effectiveness of REP in AIGC environments depends on balancing technological transparency and creative agency (Hollanek, 2023). Overly structured instruction may limit exploration, while insufficient scaffolding may impede systematic understanding (Padovano and Cardamone, 2024).

Despite its potential, REP in AIGC design education faces challenges in assessment. Evaluation must capture not only final design outcomes but also innovative thinking demonstrated during analytical deconstruction (Huang et al., 2024). A further debate concerns whether the stochastic nature of generative models undermines REP's educational validity (Bidarra and Rusman, 2017) or authentically reflects real-world design complexity, fostering adaptive creative capacity (Garib and Coffelt, 2024). Collectively, prior work establishes REP's efficacy in engineering and STEM contexts. Its application in AIGC design education, characterized by generative uncertainty, remains empirically underexamined, the gap this study addresses.

### 2.3 Learning motivation

Learning motivation, grounded in SDT, is conceptualized as a multidimensional construct comprising both intrinsic and extrinsic components (Deci and Ryan, 1985). Intrinsic motivation arises from the inherent enjoyment, interest, or satisfaction derived from the learning activity itself; in this study, it corresponds to the dimensions of interest and achievement. Extrinsic motivation is regulated by external reinforcement mechanisms (e.g., rewards, evaluations, career development); here, it is operationalized through the dimensions of career development and social recognition (Ryan and Deci, 2020). Learning motivation is recognized as a key determinant of educational outcomes, providing critical insight into the behavioral drivers of learning efficacy (Linnenbrink-Garcia and Pekrun, 2011). Intrinsic motivation and extrinsic motivation jointly influence learning outcomes and creativity (Cook and Artino, 2016; Howard et al., 2021). While intrinsic motivation is critical for sustained engagement, appropriately designed extrinsic incentives can also enhance creative performance (DePasque and Tricomi, 2015). Excessive reliance on extrinsic rewards, however, may weaken intrinsic motivation, reducing long-term engagement and diminishing creative capacity (Bear et al., 2017).

The rapid development of AIGC has positioned motivational theories as a crucial research focus in AIGC design education. AIGC-driven design pedagogy demands strong creative and technical competencies, making motivational stimulation essential for creativity enhancement (Hwang et al., 2020). Designing tasks that meet autonomy and competence needs can enhance both intrinsic and extrinsic motivation, enabling higher creative potential in AIGC-based design activities (Li et al., 2024). Thus, SDT provides a theoretical foundation for regulating student motivation in design-based and creativity-oriented educational contexts, where motivational enhancement is critical for fostering creativity (Forgeard, 2024). Within AIGC education, evidence-based pedagogical interventions that strengthen motivation hold significant potential to promote innovation and creative development (Tierney and Farmer, 2002). While SDT provides a robust framework for understanding motivational dynamics in educational settings, its application to AIGC design education requires targeted investigation, where technological novelty may simultaneously enhance perceived competence while potentially undermining intrinsic interest. This study contributes by operationalizing the four motivational dimensions of interest, achievement, career development, and social recognition as parallel mediators, thereby clarifying which specific motivational pathways are most responsive to REP within the AIGC context.

## 3 Methods

### 3.1 Participants

A quasi-experimental design with intact class-based allocation was conducted in an Animation Scene Design course at a university in Nanjing, China, to evaluate the effectiveness of REP on student creativity in AIGC-driven design education. Participants comprised 80 first-year animation majors (aged 18-20) from two intact classes, with 40 students in each class. The two classes were assigned to either an experimental group (EG) receiving REP-based intervention (*n* = 40; 18 male, 45%; 22 female, 55%) or a control group (CG) exposed to traditional instruction (*n* = 40; 19 male, 47.5%; 21 female, 52.5%) using quasi-random allocation. All participants had basic drawing and design skills but no prior formal training in animation scene design.

The experiment was supervised by a professor with over 10 years of experience in animation pedagogy. Participation was voluntary, and students could withdraw at any stage without penalty. Written informed consent was obtained following a detailed explanation of research procedures. Students were assured that questionnaire responses would remain anonymous and would not affect course evaluations or academic grades.

### 3.2 Questionnaire design

The core variables in this study comprise the independent variable (REP intervention), the mediating variable (learning motivation), and the dependent variable (creativity). To ensure reliability, validity, and theoretical consistency, all variables were measured using adapted versions of well-established domestic and international scales. The dependent variable, creativity, was assessed across four dimensions: innovativeness (INN), flexibility (FLEX), originality (ORIG), and interdisciplinary integration (II). This dimensional framework was adapted from the validated creativity scale developed by Zhou and George (2001). Their framework has been widely utilized in empirical studies on AI-enabled design education and comprehensively captures the core criteria for evaluating students' creative capabilities within AIGC collaborative creation scenarios. Based on SDT and prior literature, the mediating variable, learning motivation, was conceptualized across two levels encompassing four dimensions: intrinsic motivation [interest (INT) and achievement (ACH)] and extrinsic motivation [career development (CD) and social recognition (SR)]. The measurement items were adapted from the validated scale by Ryan and Deci (2000), a seminal instrument widely used for assessing learning motivation within the SDT framework in educational contexts. The finalized questionnaire was structured around the eight aforementioned dimensions, with two items assigned to each dimension. All items were rated on a 5-point Likert scale, ranging from 1 (strongly disagree) to 5 (strongly agree). The specific items and their corresponding sources are detailed in Table 1. Furthermore, to mitigate common method bias (CMB) and social desirability bias inherent in self-reported data, two procedural controls were implemented. First, the surveys were administered anonymously. Participants were explicitly assured that their responses were strictly for academic research and would not impact their course grades, thereby alleviating concerns and minimizing social desirability bias. Second, the presentation order of items from both the creativity and learning motivation scales was randomized to further prevent CMB.

**Table 1:** Questionnaire dimensions, items, and sources.

Variables	Dimensions	Items	Source
Creativity	INN	NN1: I am able to propose novel solutions fundamentally different from traditional designs.	Zhou and George, 2001
		NN2: my design solutions frequently incorporate creative ideas others have not considered.	
	FLEX	FLEX1: when encountering design difficulties, I experiment with multiple approaches to resolve them.	
		FLEX2: I excel at modifying existing concepts to address emerging design requirements.	
	ORIG	ORIG1: my design works exhibit distinctive personal characteristics.	
		ORIG2: others frequently describe my design outcomes as unconventional.	
	II	II1: I systematically integrate knowledge from other disciplines into design practice.	
		II2: my designs often synthesize tools/techniques from diverse domains.	
Learning motivation	INT	INT1: learning AIGC design excites me and feels intrinsically enjoyable.	Ryan and Deci, 2000
		INT2 I proactively explore new functionalities of AIGC tools.	
	ACH	ACH1: completing complex design tasks provides me with profound satisfaction.	
		ACH2: I can clearly observe my progressive improvement in AIGC design.	
	CD	CD1: mastering AIGC skills will enhance my future employment prospects.	
		CD2: my primary motivation for learning AIGC design is career advancement.	
	SR	SR1: Recognition from instructors/peers regarding my designs motivates increased effort.	
		SR2: I aspire to receive professional accolades or awards through AIGC design.	

### 3.3 Instructional implementation

#### 3.3.1 Instructional content

**Figure 2:**
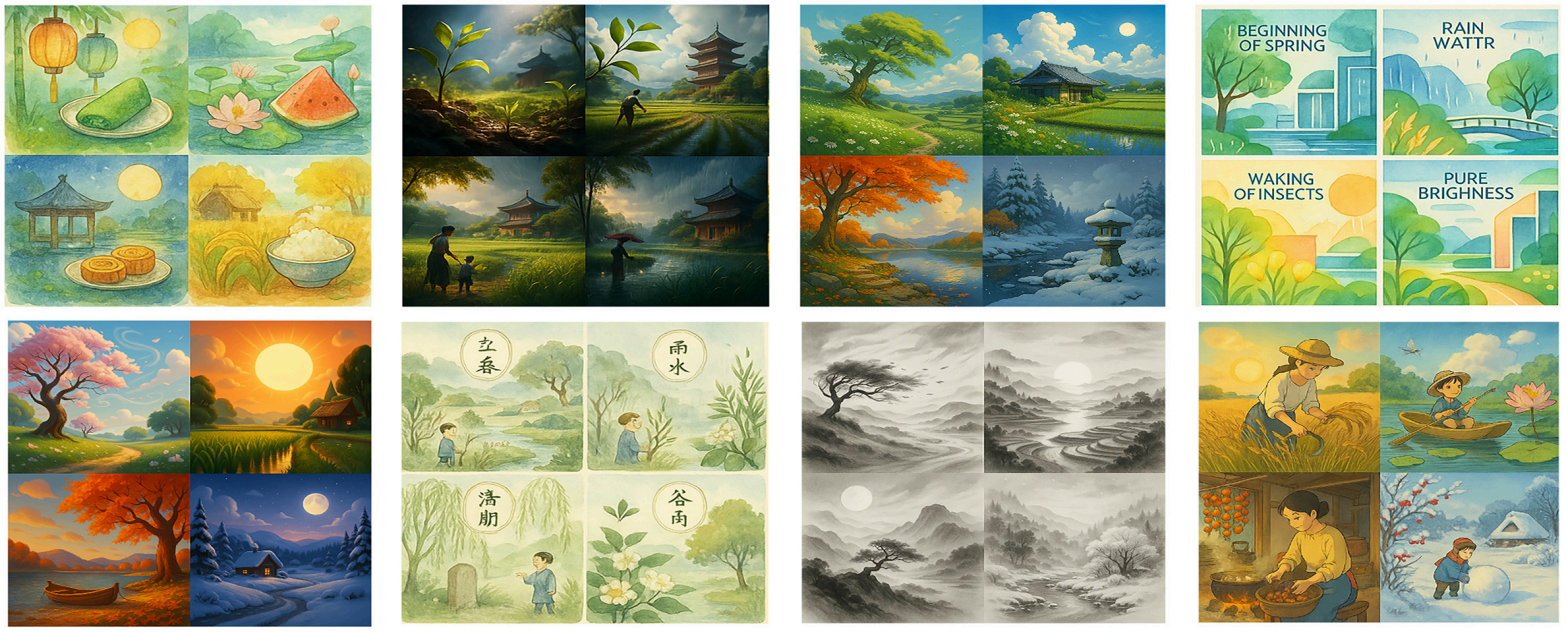
Student-generated AIGC scene design works: the 24 solar terms theme.

The experimental study focused on the traditional Chinese cultural theme of the “Twenty-Four Solar Terms,” emphasizing AIGC-enabled animated scene design (as shown in Figure 2). As a significant component of Chinese cultural heritage, the Twenty-Four Solar Terms embody the interaction between humanity and nature, encompassing natural phenomena, agricultural practices, and seasonal transitions. This theme served two primary purposes: deepening students' understanding of cultural legacy while stimulating creative cognition. Each solar term possesses distinct natural and symbolic characteristics. For example, “Lichun” (Spring Commences) symbolizes renewal and vitality, whereas “Qiufen” (Autumnal Equinox) represents harvest and fulfillment. These elements provided rich material for creative reinterpretation through AIGC technologies. During the design process, students transformed these cultural symbols into artistic visual representations, integrating abstract cultural motifs and natural landscapes with digital animation effects. The task required synthesizing traditional cultural elements with modern technological approaches, thereby fostering systematic thinking and innovative design competence within AIGC applications.

#### 3.3.2 Experimental intervention protocol

**Figure 3:**
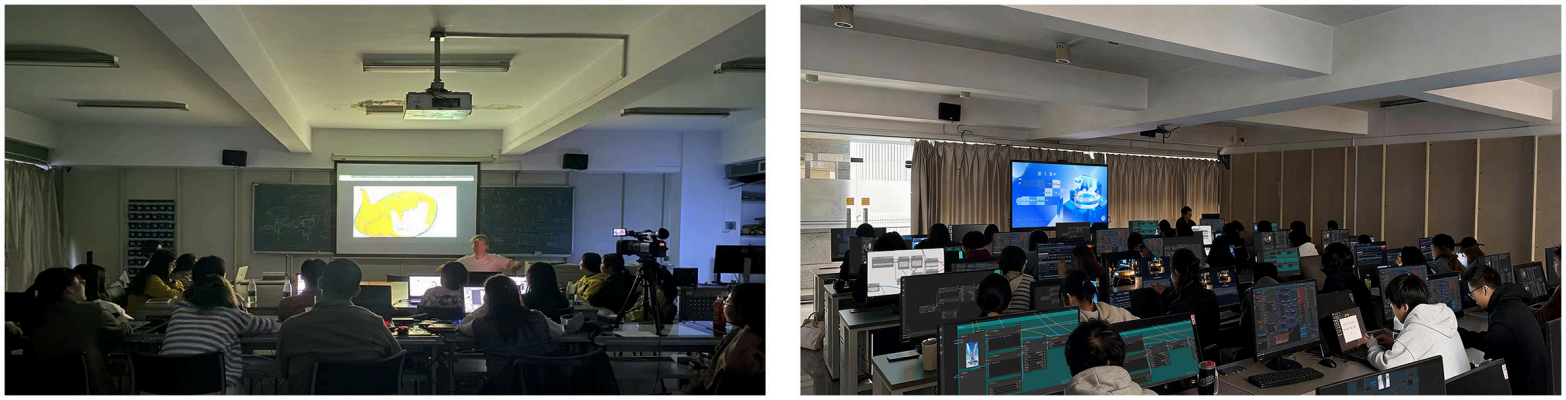
Pedagogical environment for AIGC scene design instruction.

The study compared REP-based instruction (experimental group) with conventional teaching (control group) in AIGC scene design education, and the instructional settings for both groups are shown in Figure 3. The instructional sequence for the experimental group, adapted from (Liu et al., 2023), comprised three phases: (1) structural analysis of AIGC cases, (2) functional mapping of design logic, (3) creative reconstruction of innovative solutions. Conversely, the control group's pedagogical progression transitioned from instructor-led foundational design instruction to student self-directed creation. The pedagogical intervention was conducted over an 8-week period, comprising one 4-h session per week, yielding a total of 32 instructional hours for both groups. For the experimental group, the curriculum was strictly structured around the three REP phases: Phase 1 (Weeks 1–2, 8 h) focused on structural analysis, where students systematically deconstructed exemplar AIGC cases; Phase 2 (Weeks 3–5, 12 h) centered on functional mapping, emphasizing the correlation between traditional cultural elements of the “Twenty-Four Solar Terms” and AIGC prompts; and Phase 3 (Weeks 6–8, 12 h) involved creative reconstruction, where students finalized their self-directed designs.

In the experimental group's first instructional phase, educators guided students in deconstructing structural elements of exemplary Twenty-Four Solar Terms AIGC design cases through systematic analysis of composition, color application, and design features. Simultaneously, instructors analyzed textual prompts from existing works, extracting visual element descriptors, including pictorial style and compositional frameworks. In the second phase, building on structural analysis, teachers mapped solar term themes to specific design functionalities using curated case studies. This process helped students understand how to translate natural characteristics into functional design elements. For example, the “Winter Solstice” theme was expressed through chromatic schemes and structural arrangements that conveyed a frigid atmosphere while enhancing scene interactivity. The final creative reconstruction phase required students to synthesize the deconstructed elements and design logic from previous stages. Using AIGC image-generation tools such as Stable Diffusion and Midjourney, they recontextualized traditional elements to develop personalized and innovative solutions, producing outputs characterized by high creativity.

To highlight the specific effects of the REP intervention, the control group utilized an identical “Twenty-Four Solar Terms” design theme and utilized the exact same AIGC tools (Stable Diffusion and Midjourney). However, their instruction lacked the structured analytical scaffolding characteristic of REP. Instruction began with conventional scene design principles, emphasizing foundational chromatic theory and structural composition. Subsequently, students engaged in self-directed AIGC-assisted design. Instructor guidance during this phase was strictly confined to technical troubleshooting and basic software operation, devoid of systematic case deconstruction or functional prompt mapping. Consequently, the control group's creative process relied heavily on unguided trial-and-error and individual intuition, allowing us to attribute behavioral differences squarely to the presence or absence of the REP pedagogical framework.

### 3.4 Data analysis

A hierarchical, multi-step multivariate statistical analysis was conducted to investigate variable interaction pathways. The analysis, performed in SPSS (Version 27) (IBM, Armonk, NY, USA), followed four sequential phases: reliability and validity testing, difference analysis, correlation examination, and mediation effect verification. Descriptive statistics were used to characterize variable distribution patterns. Reliability was assessed using Cronbach's α coefficients, and construct validity was evaluated through the Kaiser–Meyer–Olkin (KMO) test and Bartlett's test of sphericity. Independent samples *t*-tests were applied to compare between-group differences, while paired samples *t*-tests examined longitudinal effects of the intervention. Pearson correlation coefficients quantified the relationships between learning motivation and creativity. Multiple linear regression modeling was used to determine the direct effects of the REP. To explore underlying mechanisms, a mediation model tested the intermediary role of learning motivation. A multi-factor mediation analysis further assessed differential contributions of four motivational dimensions—interest, achievement, career development, and social recognition. This integrated analytical framework, combining parametric testing, correlation mapping, regression modeling, and mediation pathway analysis, systematically deconstructed the intervention's effects on key variables.

## 4 Results

### 4.1 Reliability and validity testing

Reliability testing assessed the internal consistency of the questionnaire. As shown in Table 2, Cronbach's α coefficients for creativity (0.773) and learning motivation (0.770) exceeded the commonly accepted threshold of 0.7, indicating strong internal consistency. Values closer to 1 denote higher inter-item correlations and measurement stability. These results confirm the reliability and psychometric soundness of the measurement instruments used in this study.

**Table 2:** Reliability testing of the questionnaire scale.

Dimensions	Items	Cronbach' α
Creativity	8	0.773
Learning motivation	8	0.77

Construct validity was evaluated using the KMO test and Bartlett's test of sphericity. As shown in Table 3, the KMO value was 0.731, indicating moderate inter-variable correlations and adequate sampling suitability for factor analysis. A KMO value above 0.7 is generally considered acceptable for extracting underlying factors. The Bartlett's sphericity test produced a chi-square value of 548.982 (*df* = 120, *p* < 0.001), confirming significant correlations among variables. Together, these results validate the structural applicability of the dataset for factor analysis, ensuring that the measurement items accurately represent the intended constructs.

**Table 3:** Validity testing of the questionnaire scale.

KMO		0.731
Bartlett's test of sphericity	χ^2^	548.982
	*df*	120
	*p*	0.000

### 4.2 Difference analysis

#### 4.2.1 Pre-test independent samples *t*-test

As shown in Table 4, the pre-test independent samples *t*-test indicated no significant differences between the control and experimental groups in either creativity or learning motivation. For creativity, the control group (*M* = 3.39, SD = 0.71) and the experimental group (*M* = 3.20, SD = 0.71) displayed comparable scores, *t*_(78)_ = 1.223, *p* = 0.225, confirming no statistically significant disparity. Similarly, learning motivation scores for the control group (*M* = 3.11, SD = 0.63) and the experimental group (*M* = 3.22, SD = 0.75) were statistically equivalent, *t*_(78)_ = −0.684, *p* = 0.496. These results confirm baseline equivalence in both variables, ensuring methodological validity for subsequent comparative analyses.

**Table 4:** Independent samples *t*-test for global variables and subdivided dimensions in pre-test and post-test.

Paired	Variables	Group (*M* ±SD)		*t*(*df*)	*p*	Dimensions	Group (*M* ±SD)		*t*(*df*)	*p*
		CG (*n* = 40)	EG (*n* = 40)				CG (*n* = 40)	EG (*n* = 40)		
Pre-test	Creativity	3.39 ± 0.71	3.20 ± 0.71	1.223 (78)	0.225	INN	3.46 ± 0.90	3.20 ± 1.09	1.173 (78)	0.244
						FLEX	3.35 ± 0.94	3.14 ± 1.01	0.978 (78)	0.331
						ORIG	3.42 ± 1.02	3.38 ± 1.03	0.218 (78)	0.828
						II	3.34 ± 1.02	3.09 ± 1.15	1.026 (78)	0.308
	Learning motivation	3.11 ± 0.63	3.22 ± 0.75	–0.684 (78)	0.496	INT	3.15 ± 0.92	3.16 ± 0.96	–0.060 (78)	0.953
						ACH	3.02 ± 1.01	3.30 ± 1.05	–1.193 (78)	0.236
						CD	3.13 ± 0.95	2.92 ± 1.18	0.837 (78)	0.405
						SR	3.14 ± 0.93	3.48 ± 0.94	–1.617 (78)	0.11
Post-test	Creativity	3.38 ± 0.47	4.02 ± 0.41	–6.376 (78)	0.000^***^	INN	3.42 ± 0.85	4.17 ± 0.75	-4.187 (78)	0.000^***^
						FLEX	3.51 ± 0.79	3.95 ± 0.92	–2.286 (78)	0.025^*^
						ORIG	3.34 ± 0.84	3.90 ± 0.89	–2.922 (78)	0.005^**^
						II	3.25 ± 0.99	4.04 ± 0.77	–3.976 (78)	0.000^***^
	Learning motivation	3.24 ± 0.51	3.92 ± 0.43	–6.526 (78)	0.000^***^	INT	3.23 ± 0.84	4.03 ± 0.82	–4.302 (78)	0.000^***^
						ACH	3.17 ± 0.58	3.94 ± 0.72	–5.214 (78)	0.000^***^
						CD	3.41 ± 0.81	3.81 ± 0.90	–2.087 (78)	0.040^*^
						SR	3.20 ± 0.76	3.91 ± 0.74	–4.250 (78)	0.000^***^

The pre-test independent samples *t*-test for subdivided dimensions also revealed no significant differences across any secondary indicators of creativity or learning motivation. Although minor variations were observed in mean scores, all p-values exceeded 0.05, confirming the absence of statistically significant disparities between the two groups. These results establish fundamental equivalence across all subdimensions, satisfying the prerequisite for valid experimental comparisons.

#### 4.2.2 Post-test independent samples *t*-test

As presented in Table 4, the post-test independent samples *t*-test demonstrated statistically significant differences between the experimental and control groups for both creativity and learning motivation. Creativity scores in the experimental group (*M* = 4.02, SD = 0.41) were significantly higher than those in the control group (*M* = 3.38, SD = 0.47), with a large effect size, *t*_(78)_ = −6.376, *p* < 0.001, Cohen's *d* = 1.45, 95% CI [0.96, 1.94]. Similarly, learning motivation scores were significantly greater in the experimental group (*M* = 3.92, SD = 0.43) compared to the control group (*M* = 3.24, SD = 0.51), *t*_(78)_ = −6.526, *p* < 0.001, Cohen's *d* = 1.44, 95% CI [0.95, 1.93]. According to Cohen's (1988) benchmarks, a *d* value of 0.2 represents a small effect, 0.5 a medium effect, and 0.8 a large effect. These findings provide strong evidence of the REP intervention's effectiveness, confirming substantial improvements in both creativity and learning motivation following the experimental treatment.

The post-test independent samples *t*-test for subdivided dimensions demonstrated that the experimental group significantly outperformed the control group across all creativity and learning motivation components (*p* < 0.05 for all indicators). The experimental group achieved higher scores in innovativeness, flexibility, originality, and interdisciplinary integration, as well as in interest, achievement, career development, and social recognition. These consistent improvements across all measured dimensions confirm the effectiveness of the REP pedagogical intervention in fostering multidimensional creativity and learning motivation outcomes.

#### 4.2.3 Control group paired samples *t*-test

As shown in Table 5, paired samples *t*-test results for the control group indicated no statistically significant differences between pre-test and post-test scores for either creativity or learning motivation. Creativity scores showed minimal change between pre-test (*M* = 3.39, SD = 0.71) and post-test (*M* = 3.38, SD = 0.47, MD = 0.01), *t*_(39)_ = 0.097, *p* = 0.923. Learning motivation scores also remained stable, with no significant difference between pre-test (*M* = 3.11, SD = 0.63) and post-test (*M* = 3.24, SD = 0.51, MD = –0.13), *t*_(39)_ = −1.112, *p* = 0.273. The control group showed no significant pre-to-post change in creativity (MD = 0.01, *t*_(39)_ = 0.097, *p* = 0.923, Cohen's *d* = 0.02) or learning motivation (MD = –0.13), *t*_(39)_ = −1.112, *p* = 0.273, Cohen's *d* = 0.18. Variance ratio analysis confirmed a significant reduction change in creativity score variance between pre-test (SD = 0.71) and post-test (SD = 0.47; *F* = 2.276, *p* = 0.008). However, this variance reduction was not accompanied by a significant mean improvement, indicating that students' performance became more homogeneous but remained at a low level without systematic creative growth. The gain score distribution analysis revealed that approximately 65% of control participants had creativity gains below 0.30, and no participant achieved a gain exceeding 1.00. This authentically reflects the risk of AIGC integration without carefully designed instructional guidance; mere exposure to tools without structured pedagogical scaffolding leads to routine application rather than meaningful creative development. The significant improvement observed in the experimental group further supports this interpretation, indicating that a structured REP intervention, rather than simply providing tools, is the key driver of creativity development.

**Table 5:** Paired samples *t*-test for global variables and subdivided dimensions in control group and experimental group.

Group	Variables	Paired (*M* ±SD)		MD	*t*(*df*)	*p*	Dimensions	Paired (M ±SD)		MD	*t*(*df*)	*p*
		Pre-test	Post-test					Pre-test	Post-test			
CG	Creativity	3.39 ± 0.71	3.38 ± 0.47	0.01	0.097 (39)	0.923	INN	3.46 ± 0.90	3.42 ± 0.85	0.04	0.194 (39)	0.847
							FLEX	3.35 ± 0.94	3.51 ± 0.79	–0.16	–0.906 (39)	0.371
							ORIG	3.42 ± 1.02	3.34 ± 0.84	0.09	0.403 (39)	0.689
							II	3.34 ± 1.02	3.25 ± 0.99	0.09	0.371 (39)	0.713
	Learning motivation	3.11 ± 0.63	3.24 ± 0.51	–0.13	–1.112 (39)	0.273	INT	3.15 ± 0.92	3.23 ± 0.84	–0.08	–0.369 (39)	0.714
							ACH	3.02 ± 1.01	3.17 ± 0.58	–0.15	–0.966 (39)	0.34
							CD	3.13 ± 0.95	3.41 ± 0.81	–0.29	-1.431 (39)	0.16
							SR	3.14 ± 0.93	3.20 ± 0.76	–0.06	–0.320 (39)	0.751
EG	Creativity	3.20 ± 0.71	4.02 ± 0.41	–0.82	–5.161 (39)	0.000^***^	INN	3.20 ± 1.09	4.17 ± 0.75	–0.97	-4.106 (39)	0.000^***^
							FLEX	3.14 ± 1.01	3.95 ± 0.92	–0.81	-3.536 (39)	0.001^**^
							ORIG	3.38 ± 1.03	3.90 ± 0.89	–0.52	-2.243 (39)	0.031^*^
							II	3.09 ± 1.15	4.04 ± 0.77	–0.95	-3.852 (39)	0.000^***^
	Learning motivation	3.22 ± 0.75	3.92 ± 0.43	–0.71	–4.848 (39)	0.000^***^	INT	3.16 ± 0.96	4.03 ± 0.82	–0.86	-4.101 (39)	0.000^***^
							ACH	3.30 ± 1.05	3.94 ± 0.72	–0.64	-3.148 (39)	0.003^**^
							CD	2.92 ± 1.18	3.81 ± 0.90	–0.89	-3.938 (39)	0.000^***^
							SR	3.48 ± 0.94	3.91 ± 0.74	–0.44	-2.229 (39)	0.032^*^

^*^p < 0.05; ^**^p < 0.01; ^***^p < 0.001.

Paired samples *t*-test results for subdivided dimensions revealed no statistically significant differences between pre-test and post-test scores across any creativity or learning motivation components within the control group. For creativity, no significant differences were found in INN (*p* = 0.847), FLEX (*p* = 0.371), ORIG (*p* = 0.689), or II (*p* = 0.713). Likewise, learning motivation components, including INT (*p* = 0.714), ACH (*p* = 0.340), CD (*p* = 0.160), and SR (*p* = 0.751), showed no significant variation. These results demonstrate stability across all measured indicators in the control group, confirming the absence of spontaneous developmental changes during the experimental timeline.

#### 4.2.4 Experimental group paired samples *t*-test

As shown in Table 5, the paired samples *t*-test results for the experimental group indicated significant improvements in both creativity and learning motivation following the intervention. Creativity scores increased from 3.20 ± 0.71 (pre-test) to 4.02 ± 0.41 (post-test), with a mean difference of –0.82, *t*_(39)_ = −5.161, *p* < 0.001, Cohen's *d* = 0.82. Learning motivation similarly improved, rising from 3.22 ± 0.75 to 3.92 ± 0.43, with a mean difference of –0.71, *t*_(39)_ = −4.848, *p* < 0.001, Cohen's *d* = 0.77. These results confirm that the REP intervention produced significant positive effects on both variables.

Notably, the standard deviation of creativity scores in the experimental group decreased significantly from 0.71 (pre-test) to 0.41 (post-test). To provide a more granular empirical validation of the effectiveness of the REP intervention, a subgroup analysis was performed. The experimental cohort (*n* = 40) was categorized into two equal subgroups based on a median split of their pre-test creativity scores: a low-baseline subgroup (*n* = 20) and a high-baseline subgroup (*n* = 20). Separate paired-samples *t*-tests were executed for each subgroup to contrast their pre- and post-intervention creative performance. For the low-baseline subgroup, creative performance escalated robustly from the pre-test (*M* = 2.63, SD = 0.45) to the post-test (*M* = 3.64, SD = 0.38). This longitudinal leap was highly significant, *t*_(19)_ = −6.023, *p* < 0.001, yielding a large effect size (Cohen's *d* = 1.35), which reflects a substantial educational gain (MD = –1.01). In comparison, the high-baseline subgroup also demonstrated a statistically significant enhancement from the pre-test (*M* = 3.77, SD = 0.42) to the post-test (*M* = 4.40, SD = 0.35), *t*_(19)_ = −3.892, *p* < 0.001, yielding a medium-to-large effect size (Cohen's *d* = 0.87), accompanied by a smaller absolute growth magnitude (MD = –0.63). The difference in gain scores between the two subgroups was statistically meaningful, confirming that REP exerts a differential effect based on baseline creative ability. Ceiling effect checks showed that only two students (5%) of participants reached the maximum 5-point score in the post-test, confirming that the observed improvement was not constrained by a ceiling effect. These results statistically validate that REP effectively raised the floor for lower-performing students while sustaining growth for higher performers. The scaffolded three-phase structure provided necessary support for students who initially struggled with AIGC's technical complexity, enabling them to achieve baseline creative competency. Meanwhile, high-performing students continued to excel without being limited by the assessment scale, demonstrating that REP functions as an equity-promoting intervention that reduces performance variance without suppressing top performers.

Paired samples *t*-test results for subdivided dimensions revealed significant pretest-posttest improvements across all components of creativity and learning motivation in the experimental group. Within creativity, INN (*p* = 0.000), FLEX (*p* = 0.001), ORIG (*p* = 0.031), and II (*p* = 0.000) indicated statistically significant gains. Learning motivation subdimensions also improved significantly, including INT (*p* = 0.000), ACH (*p* = 0.003), CD (*p* = 0.000), and SR (*p* = 0.032). These results demonstrate that the REP intervention effectively enhanced all measured aspects of creativity and motivation.

### 4.3 Correlation analysis

As presented in Table 6, Pearson's correlation analysis revealed a strong positive relationship between learning motivation and creativity. The correlation coefficient (*r* = 0.703, *p* < 0.001) indicates that higher learning motivation is strongly associated with higher creativity levels. The p-value below 0.05 confirms statistical significance, and the sample size (*n* = 80) provides sufficient statistical power to support the conclusion. At the subdimensional level, most of the creativity dimensions showed significant positive correlations with the learning motivation dimensions, though the strength and significance of these associations varied across specific pairs. For example, innovativeness correlated most strongly with achievement (*r* = 0.416, *p* < 0.001) and social recognition (*r* = 0.402, *p* < 0.001), originality showed a relatively strong association with career development (*r* = 0.424, *p* < 0.001), and interdisciplinary integration was most closely linked with interest (*r* = 0.432, *p* < 0.001) and achievement (*r* = 0.389, *p* < 0.001). These results demonstrate that learning motivation is a significant predictor of creativity.

**Table 6:** Correlation test between learning motivation and creativity dimensions.

Variable	INN	FLEX	ORIG	II	INT	ACH	CD	SR	Creativity	Learning motivation
INN	1									
FLEX	0.171	1								
ORIG	0.126	0.045	1							
II	0.287^**^	0.091	0.166	1						
INT	0.219	0.101	0.356^**^	0.432^***^	1					
ACH	0.416^***^	0.178	0.218	0.389^***^	0.370^***^	1				
CD	0.243^*^	0.151	0.424^***^	0.209	0.175	0.352^**^	1			
SR	0.402^***^	0.335^**^	0.237^*^	0.279^*^	0.207	0.332^**^	0.275^*^	1		
Creativity	0.652^***^	0.531^***^	0.555^***^	0.664^***^	0.468^***^	0.502^***^	0.427^***^	0.519^***^	1	
Learning motivation	0.464^***^	0.277^*^	0.460^***^	0.483^***^	0.663^***^	0.739^***^	0.665^***^	0.656^***^	0.703^***^	1

^*^p < 0.05; ^**^p < 0.01; ^***^p < 0.001.

### 4.4 Relationship testing

#### 4.4.1 In AIGC design education, does the REP pedagogical intervention significantly enhance students' creativity?

A linear regression analysis was conducted with the REP pedagogical intervention as the independent variable and creativity as the dependent variable. As shown in Table 7, the regression equation was Creativity = 2.747 + 0.634 × REP Intervention. The coefficient of determination (*R*^2^ = 0.343) indicates that the REP intervention explains 34.3% of the variance in creativity. The model was statistically significant, *F*_(1, 78)_ = 40.655, *p* < 0.001. The REP intervention demonstrated a significant positive effect on creativity, with a regression coefficient of 0.634 (*t* = 6.376, *p* < 0.001). These findings confirm that the REP pedagogical intervention substantially enhances students' creative performance.

**Table 7:** Regression analysis of the relationship between REP and creativity.

	Unstandardized coefficients		Standardized coefficients	*t*	*p*
	*B*	SE	β		
Constant	2.747	0.157	-	17.462	0.000^***^
REP intervention	0.634	0.099	0.585	6.376	0.000^***^
*R* ^2^	0.343				
Adjusted *R*^2^	0.334				
*F*	*F*_(1, 78)_ = 40.655, *p* = 0.000				

#### 4.4.2 In AIGC design education, does the REP pedagogical intervention significantly enhance students' learning motivation?

A linear regression analysis was conducted with the REP pedagogical intervention as the independent variable and learning motivation as the dependent variable. As presented in Table 8, the regression equation was Learning Motivation = 2.553 + 0.684 × REP Intervention. The coefficient of determination (*R*^2^ = 0.353) indicates that the REP intervention accounts for 35.3% of the variance in learning motivation. The model was statistically significant, *F*_(1, 78)_ = 42.551, *p* < 0.001. The REP intervention exhibited a significant positive effect, with a regression coefficient of 0.684 (*t* = 6.523, *p* < 0.001), confirming its effectiveness in enhancing students' learning motivation.

**Table 8:** Regression analysis of the relationship between REP and learning motivation.

	Unstandardized coefficients		Standardized coefficients	*t*	*p*
	*B*	SE	β		
Constant	2.553	0.166	-	15.951	0.000^***^
REP intervention	0.684	0.105	0.594	6.523	0.000^***^
*R* ^2^	0.353				
Adjusted *R*^2^	0.345				
*F*	*F*_(1, 78)_ = 42.551, *p* = 0.000				

#### 4.4.3 Does learning motivation (interest, achievement, career development, and social recognition) play a mediating role between REP and creativity enhancement in AIGC design education?

Mediation analysis confirmed that learning motivation partially mediates the relationship between REP and creativity enhancement. As shown in Table 9, REP directly influenced creativity (*B* = 0.634, β = 0.585, *p* < 0.001) and significantly affected learning motivation (*B* = 0.684, β = 0.594, *p* < 0.001). Learning motivation, in turn, had a significant positive effect on creativity (*B* = 0.516, β = 0.549, *p* < 0.001). These findings indicate that REP enhances creativity both directly and indirectly by first increasing learning motivation, which then amplifies creative performance. The mediation pathway confirms a partial mediating effect of learning motivation in the REP-creativity relationship.

**Table 9:** Mediation effect analysis of learning motivation.

	Creativity		Learning motivation		Creativity	
	B	β	*B*	β	*B*	β
Constant	2.747^***^	-	2.553^***^	-	1.429^***^	-
REP intervention	0.634^***^	0.585	0.684^***^	0.594	0.281^**^	0.259
Learning motivation					0.516^***^	0.549
*R* ^2^	0.343		0.353		0.537	
Adjusted *R*^2^	0.334		0.345		0.525	
*F*	40.655^***^		42.551^***^		44.733^***^	

#### 4.4.4 How can a multidimensional creativity evaluation framework suitable for AIGC design education be proposed based on REP and learning motivation?

A multiple regression analysis was conducted to assess the effects of four learning motivation dimensions on creativity. As shown in Table 10, all four dimensions significantly predicted creativity, with varying effect sizes. SR demonstrated the strongest positive effect (β = 0.332, *p* < 0.001), followed by interest (β = 0.283, *p* = 0.002). ACH (β = 0.213, *p* = 0.028) and CD (β = 0.211, *p* = 0.020) had weaker yet significant effects. Collinearity diagnostics confirmed the absence of serious multicollinearity issues (VIF < 1.5; Tolerance > 0.7), indicating that each dimension exerted independent effects on creativity. These findings highlight the differential contributions of motivational dimensions, suggesting that social recognition and interest are primary drivers of creativity, while achievement and career development provide supportive but less pronounced contributions.

**Table 10:** Effects of learning motivation dimensions on creativity.

	Unstandardized coefficients		Standardized coefficients	*t*	*p*	Collinearity diagnostics	
	*B*	SE	β			VIF	Tolerance
Constant	1.32	0.279	-	4.737	0.000^***^	-	-
INT	0.168	0.052	0.283	3.211	0.002^**^	1.171	0.854
ACH	0.147	0.065	0.213	2.244	0.028^*^	1.351	0.74
CD	0.131	0.055	0.211	2.38	0.020^*^	1.181	0.846
SR	0.219	0.058	0.332	3.759	0.000^***^	1.17	0.855
*R* ^2^	0.501						
Adjusted *R*^2^	0.475						
*F*	*F*_(4, 75)_ = 18.849, *p* = 0.000						

^*^p < 0.05, ^**^p < 0.01, ^***^p < 0.001.

The multiple mediation analysis confirmed that the four dimensions of learning motivation partially mediate the relationship between REP and creativity enhancement, including social recognition, interest, career development, and achievement. As shown in Table 11, all four dimensions significantly mediated the REP-creativity relationship, although their contributions varied. In the REP → INT → Creativity pathway, the total effect was 0.634, with an indirect effect of 0.124 and a direct effect of 0.510. The mediating effect accounted for 19.56% of the total influence. In the REP → ACH → Creativity pathway, the total effect was 0.634, with an indirect effect of 0.153 and a direct effect of 0.482. The mediation accounted for 24.08%, representing the largest share among all dimensions. In the REP → CD → Creativity pathway, the total effect was 0.634, with an indirect effect of 0.077 and a direct effect of 0.557. The mediating effect accounted for 12.12%, indicating the smallest contribution. In the REP → SR → Creativity pathway, the total effect was 0.634, with an indirect effect of 0.153 and a direct effect of 0.481. The mediation accounted for 24.19%, similar to the contribution of ACH. Overall, these results indicate that REP enhances creativity both directly and indirectly through the mediating influence of learning motivation. Achievement and social recognition contribute the most to this mediating pathway, underscoring their pivotal roles in the effectiveness of REP for creativity enhancement.

**Table 11:** Mediation path analysis of the four dimensions in the relationship between REP and creativity.

Mediation path	*c*	a × b	c'	Proportion mediated	Conclusion
	Total effect	Indirect effect	Direct effect		
REP → INT → Creativity	0.634	0.124	0.51	0.1956	Partial mediation
REP → ACH → Creativity	0.634	0.153	0.482	0.2408	Partial mediation
REP → CD → Creativity	0.634	0.077	0.557	0.1212	Partial mediation
REP → SR → Creativity	0.634	0.153	0.481	0.2419	Partial mediation

## 5 Discussion

This study used a quasi-experimental design to evaluate the effectiveness of REP in enhancing creativity within AIGC-based design education. It also examined the mediating role of learning motivation and established a multidimensional framework for creativity evaluation. The results demonstrate that REP significantly improves students' creative performance and indirectly promotes creativity development by stimulating learning motivation. These findings validate REP as an effective instructional approach in AIGC design education and provide both theoretical justification and practical strategies for educational implementation. By integrating technological tools, pedagogical strategies, and psychological mechanisms, the study offers new methodological perspectives for cultivating creativity among students engaged in AIGC design education.

### 5.1 In AIGC design education, does the REP pedagogical intervention significantly enhance students' creativity?

The results confirm that REP has a significant positive effect on student creativity in AIGC-based design education. The experimental group scored significantly higher than the control group in all creativity dimensions during the post-test, consistent with prior findings on the effectiveness of REP in traditional engineering education (Ladachart et al., 2022). This study extends REP's application by incorporating AIGC-generated design cases. Students gained insight into the technical logic underlying AIGC design processes and moved beyond imitative learning to develop original design thinking. REP's unique advantage in AIGC education lies in its ability to visually and intuitively demonstrate the operational logic of AI technologies (Hollanek, 2023). This transparency is crucial because AIGC tools have reconfigured the creative process: whereas traditional design education focused on cultivating divergent ideation skills, AIGC environments demand enhanced capabilities for evaluative critique and reconstructive synthesis. When students reverse-engineer AI-generated cases, they practice precisely these redefined creative competencies: discerning quality among algorithmic variations, identifying semantic gaps between prompts and outputs, and purposefully recombining elements into culturally meaningful compositions. Understanding these mechanisms reduces students' uncertainty regarding generative models, enabling them to extract design principles from AI-generated cases and apply them flexibly when adjusting their design strategies. The process of identifying discrepancies between AI-generated outputs and personal expectations also compelled students to reconstruct existing knowledge frameworks to interpret these unexpected results (Cascini et al., 2022). This knowledge reconstruction directly activated divergent thinking and enhanced problem-solving ability.

The large effect sizes observed in this study warrant further interpretation. Several factors may account for the pronounced impact of REP on creativity. First, the REP intervention represented a fundamental difference in instructional design: the experimental group received systematic training in higher-order cognitive skills (structural analysis, functional mapping, and creative reconstruction), whereas the control group received only technical instruction in tool operation. Second, AIGC technology eliminated the technical barriers that typically dilute intervention effects in traditional design education, enabling students to directly translate their improved cognitive strategies into creative outputs. Third, the participants were first-year students without prior formal training in design thinking or AIGC tools, resulting in substantial initial learning gains from structured instruction. These contextual factors also highlight the boundary conditions under which REP is most effective. Unlike traditional project-based instruction and previous studies that mainly emphasize the optimization of tool functionality (Bond et al., 2024), the theoretical contribution of this research lies in clarifying the interaction between pedagogical approaches and technological features. The proposed three-phase instructional framework: structural analysis, functional mapping, and creative reconstruction, demonstrates how to balance technological assistance with autonomous innovation. This balance helps students avoid overreliance on AI tools and prevents blind trial-and-error learning, resulting in enhanced creative performance.

### 5.2 Does learning motivation (interest, achievement, career development, and social recognition) play a mediating role between REP and creativity enhancement in AIGC design education?

The results confirm that learning motivation partially mediates the relationship between REP and creativity, indicating that REP enhances creativity both directly and indirectly by stimulating intrinsic and extrinsic motivation. This aligns with SDT, which posits that effective instructional interventions satisfy students' needs for autonomy, competence, and relatedness (Ryan and Deci, 2020). To clarify the underlying psychological mechanism, the three REP phases can be explicitly mapped to the satisfaction of SDT's basic needs: structural analysis demystifies AIGC workflows and builds perceived competence; collaborative functional mapping fosters a sense of shared inquiry and relatedness; and creative reconstruction empowers students with informed, self-directed choices, thereby fulfilling autonomy. This scaffolded progression ensures that the freedom exercised in creation is both purposeful and psychologically grounded. Compared with the AI education motivation model proposed by Xia et al. (2022), this study integrates REP's structured task design with motivational mechanisms, revealing a dynamic balance between technical implementation and creative freedom. These findings provide practical implications for AIGC design education. Phased feedback mechanisms, such as interim project presentations, can enhance extrinsic motivation through social recognition, while open-ended design problems sustain intrinsic exploratory drive.

The mediating role of career development motivation highlights REP's unique relevance in AIGC education. Students perceive REP as a pathway to acquiring advanced skills in the AIGC design industry, and this perceived career value reinforces innovative behavior. Unlike traditional STEM education, which emphasizes intrinsic interest as the primary motivational driver (Howard et al., 2021), this study proposes a “Technology-Motivation-Creativity” linkage model, emphasizing the combined influence of cognitive growth and career aspirations. For instructional design, educators should encourage reverse engineering of commercial-grade AI design cases, aligning skill acquisition with professional contexts. Such integration strengthens the mediating effect of learning motivation and further enhances creativity in AIGC-based design education.

### 5.3 How can a multidimensional creativity evaluation framework suitable for AIGC design education be proposed based on REP and learning motivation?

The theoretical framework of this study is grounded in SDT, which emphasizes the synergistic roles of intrinsic and extrinsic motivation in fostering creativity. While previous research has confirmed the effectiveness of REP in enhancing student creativity (Liu et al., 2023) and highlighted the importance of multidimensional assessment in design education (Hasibuan and Azizah, 2023), no prior work has integrated empirically validated motivational mediation mechanisms into creativity evaluation frameworks tailored to AIGC contexts. This study addresses this critical gap by proposing a motivation-mediated multidimensional creativity evaluation framework.

**Figure 4:**
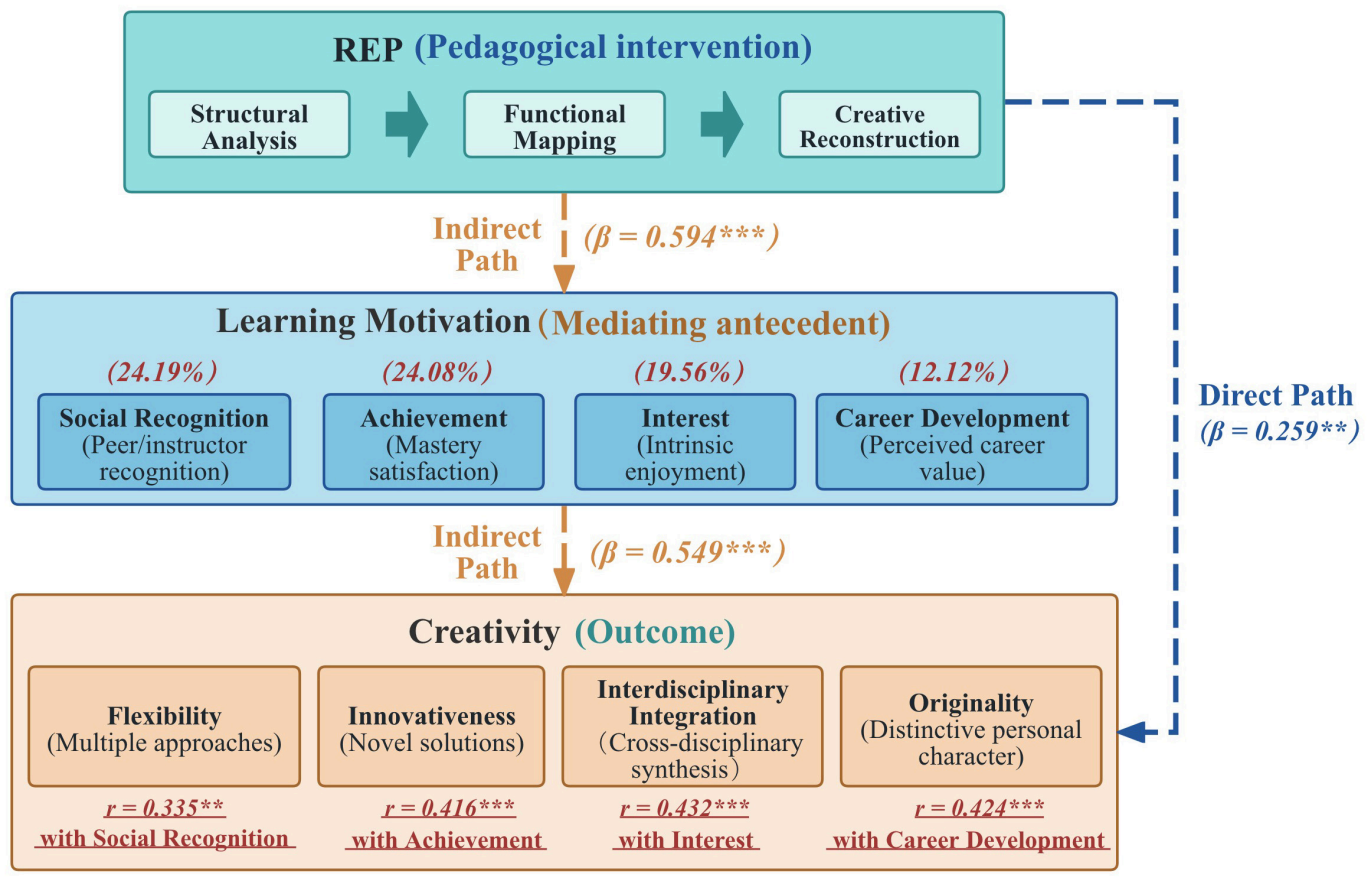
Multidimensional creativity evaluation framework for AIGC design education. Green solid arrows indicate the sequential three-phase structure of the REP. Orange and blue dashed arrows represent indirect and direct paths, with standardized regression coefficients (β) shown; values in parentheses denote the mediation proportion for each motivational dimension; *R*-values indicate Pearson correlation coefficients between the corresponding dimensions. ^**^p < 0.01; ^***^p < 0.001.

Building on the partial mediation model verified in this study, the framework is structured around three sequentially linked causal layers. As shown in Figure 4, the first layer is the pedagogical intervention layer, comprising the three-phase REP structure of structural analysis, functional mapping, and creative reconstruction. This layer serves as the external instructional input that initiates the learning process. The second layer is the motivational antecedent layer, consisting of four dimensions of learning motivation that function as psychological mediators. Collectively, these dimensions mediate a substantial proportion of REP's total effect on creativity, with varying degrees of contribution across dimensions. The third layer is the creative outcome layer, encompassing four dimensions of creativity that represent the ultimate educational objectives of AIGC design education. This framework positions learning motivation not as a parallel process indicator, but as a measurable intermediate causal node that connects pedagogical intervention to creative outcomes. REP exerts its influence via two concurrent pathways: an indirect motivational mediation pathway, through which REP activates students' learning motivation to enhance their creative performance, and a direct cognitive pathway through which REP improves creativity independently of the measured motivational dimensions. The direct pathway operates through the cognitive skills training embedded in each phase of REP: structural analysis cultivates systematic deconstruction ability, functional mapping develops conceptual connection ability, and creative reconstruction enhances synthetic innovation ability. This dual-path structure dictates that a comprehensive creativity evaluation framework must assess both the cognitive skills developed through direct training and the motivational states activated through indirect stimulation. Evaluating only final creative outcomes would obscure the differential contributions of these two pathways, leading to an incomplete understanding of students' learning progress.

Within this framework, each layer consists of empirically validated dimensions with distinct assessment purposes, and the cross-layer relationships between dimensions reveal the targeted mechanisms through which REP enhances specific facets of creativity. The motivational antecedent layer comprises four dimensions that REP can systematically activate through its three-phase structure: Interest assesses the degree of intrinsic enjoyment and proactive exploration associated with AIGC design tasks; Achievement evaluates the sense of mastery and satisfaction derived from completing complex design work and observing personal progress; Career development measures the perceived instrumental value of AIGC skills for future professional advancement; Social recognition assesses the drive to gain affirmation from peers or instructors and to achieve professional distinction. The creative outcome layer comprises four dimensions that capture the core competencies of AIGC design education: Innovativeness evaluates the capacity to propose solutions that depart markedly from conventional design templates; Flexibility assesses adaptive problem-solving when students encounter unexpected design challenges; Originality captures the uniqueness and unconventionality of design outputs as evaluated by others; Interdisciplinary integration measures the ability to synthesize knowledge, methods, and tools from multiple domains within design practice. Each motivational dimension is associated with specific creativity dimensions in distinct and theoretically interpretable patterns. Interest exhibits the strongest association with interdisciplinary integration, consistent with the expectation that intrinsic curiosity drives boundary-spanning cognitive engagement. Achievement motivation shows the most robust link to innovativeness, suggesting that perceived competence growth encourages students to move beyond familiar design conventions. Career development motivation is uniquely associated with originality, indicating that students who adopt a professional perspective are more inclined to differentiate their work from existing models. Social recognition emerges as the most influential motivational predictor of overall creativity and is robustly correlated with both innovativeness and flexibility. These differentiated associations establish the diagnostic foundation of the framework, enabling educators to map specific creative weaknesses to corresponding motivational deficits. In contrast, the direct cognitive pathway exerts a broad and uniform positive effect on all four creativity dimensions by providing students with the analytical and synthetic skills necessary to translate motivational engagement into tangible creative outputs.

In summary, this framework provides educators with a systematic approach to assessing and nurturing student creativity in AIGC-enabled design education. In AIGC contexts, the generated content of artifacts often obscures the underlying cognitive and motivational processes that contributed to their production; therefore, assessing motivational antecedents provides critical insights into the conditions that enabled meaningful creative engagement. The dual-path structure guides comprehensive instructional design: educators should implement the three-phase REP structure to build students' cognitive skills through the direct pathway, while simultaneously employing targeted strategies to activate specific motivational dimensions through the indirect pathway. For instance, a deficit in originality would suggest the need to activate career development motivation by situating design briefs within professional portfolio contexts, while a deficit in interdisciplinary integration would point toward interest-enhancing strategies such as open-ended exploration tasks that reduce emphasis on graded evaluation. Teachers can use the diagnostic results from the framework to design personalized motivational interventions: for students with low social recognition motivation, increase peer review sessions and public presentation opportunities; for students with low achievement motivation, break down complex design tasks into smaller, achievable milestones with clear progress feedback; for students with low interest motivation, introduce cross-disciplinary AIGC application cases to expand their exploratory horizons. This comprehensive approach helps educators balance technological empowerment and creative autonomy, addressing the pervasive challenge of over-reliance on AIGC tools in contemporary design education.

Accordingly, the main findings of this study can be summarized as follows:

1) REP significantly enhances student creativity in AIGC design education. Students in the experimental group outperformed those in the control group across all dimensions of creativity. REP helps overcome the limitations of imitative learning and fosters original design thinking.
2) Learning motivation partially mediates the relationship between REP and creativity. Among the motivational dimensions, social recognition and achievement showed the strongest mediating effects, suggesting that REP indirectly promotes creativity by stimulating both intrinsic and extrinsic motivation.
3) Social recognition and interest are the two most significant factors influencing creativity. This finding aligns with SDT, which emphasizes the role of intrinsic motivation in fostering creativity. Although achievement and career development motivation had weaker effects, they still exerted positive influences, indicating that creativity is shaped not only by immediate interest but also by feedback from achievements and alignment with career goals.
4) A creativity evaluation framework structured around three sequentially linked layers of pedagogical intervention, motivational antecedents, and creative outcomes was proposed. This framework positions learning motivation as a mediating mechanism that transmits the effect of REP on creativity, specifying that the quality of creative engagement serves as a driving condition for the novelty and quality of the final design output.

## 6 Conclusion

Based on the research findings and discussions, this study makes three interconnected contributions to the field of AIGC design education. First, it provides systematic empirical validation of REP as an effective instructional approach for fostering creativity in AIGC-enabled learning environments, demonstrating that REP not only improves overall creative performance but also cultivates the precise competencies required for human-AI collaborative creativity: evaluative judgment, purposeful selection, and reconstructive synthesis. At a time when generative AI has fundamentally transformed the nature of creative production by assuming the computational burden of divergent generation. This finding challenges concerns that AIGC tools may diminish human creativity and instead demonstrates that structured pedagogical interventions can redirect human creative effort toward higher-order cognitive processes. Second, it advances theoretical understanding by revealing the dual-path mechanism through which REP enhances creativity: a direct cognitive training path that builds analytical and synthetic skills through REP's three-phase structure, and an indirect motivational mediation path that activates multidimensional learning motivation to drive creative engagement. This finding extends SDT to the AIGC context by demonstrating the synergistic effects of intrinsic and extrinsic motivational factors in technology-rich learning environments, and it clarifies the psychological mechanisms that link instructional design to creative outcomes. Third, it proposes a motivation-mediated multidimensional creativity evaluation framework. This framework addresses a critical limitation of traditional assessment methods, which are increasingly inadequate in AIGC contexts where the visual quality of AI-generated outputs often obscures the underlying quality of student thinking. Collectively, these contributions fill gaps in both instructional methods and evaluation systems for AIGC design education, providing a theoretically grounded and empirically validated foundation for reimagining creative education in the age of generative AI.

Despite these contributions, several limitations remain. First, the sample was relatively limited, involving only 80 first-year animation majors from a single university. This homogeneous sample restricts the external validity of the findings, and caution should be exercised when generalizing the results to other design disciplines, different academic levels, or diverse institutional contexts. The control group's stagnant scores, while potentially explainable by the novelty of technology in unstructured AIGC learning, would benefit from validation across multiple institutions to rule out site-specific factors such as institutional culture, prior student preparation, or instructor characteristics. Additionally, the short experimental duration and absence of long-term tracking of behavioral changes may limit the generalizability of the findings regarding the sustainability of REP effects. Future research should expand the sample to include students from broader disciplinary backgrounds, including visual communication, product design, digital media, and engineering design, to examine whether REP is equally effective across different design domains. Multi-site studies involving universities with varying levels of technological infrastructure and pedagogical traditions would further strengthen the generalizability of the results. Longitudinal designs with extended intervention periods and follow-up assessments at 3-month and 6-month intervals would help verify the long-term retention of creative skills and motivational changes induced by REP. Second, the study relied exclusively on quantitative self-report measures to assess both creativity and learning motivation. Although procedural controls, including anonymous survey administration and randomization of item order, were implemented to mitigate CMB and social desirability bias, these biases cannot be completely eliminated. Self-reported creativity may be influenced by students' self-perception biases, and self-reported motivation may not always correspond to actual behavioral engagement. The current findings would therefore benefit from behavioral validation using multi-source data triangulation. Future research should incorporate objective behavioral measures such as AIGC tool operation logs, design iteration counts, and blind instructor ratings of final creative products. Peer evaluations of creativity and classroom observation protocols could also provide additional perspectives on student engagement and creative performance. Integrating these diverse data sources would reduce reliance on self-reports and enhance the objectivity and robustness of the findings. Third, the study focused mainly on image-generation AIGC tools and did not examine multimodal AI technologies such as text or video generation. Future research should broaden the scope by comparing different AIGC tools to explore their respective mechanisms of influence on student creativity, thereby deepening and expanding this line of inquiry. Additionally, the study did not investigate potential moderating variables such as prior AIGC experience, creative self-efficacy, or learning styles, which may influence the effectiveness of REP interventions. Future studies could explore these boundary conditions to identify for whom and under what conditions REP is most effective.

## Data Availability

The raw data supporting the conclusions of this article will be made available by the authors, without undue reservation.
